# DNA Methylation of Tumor Suppressive miRNAs in Non-Hodgkin’s Lymphomas

**DOI:** 10.3389/fgene.2012.00233

**Published:** 2012-11-08

**Authors:** Rita Lok-Hay Yim, Yok Lam Kwong, Kwan Yeung Wong, Chor Sang Chim

**Affiliations:** ^1^Department of Medicine, Queen Mary Hospital, The University of Hong KongHong Kong, China

**Keywords:** miRNA, tumor suppressor, DNA methylation, lymphoma

## Abstract

DNA methylation is an epigenetic alteration leading to heritable phenotypic changes of cells with functional consequences. It is important in early embryonic development, stem cell differentiation, and tissue-specific gene expression. In normal cells, promoter-associated CpG islands (CGI) are generally unmethylated except in X-chromosome inactivation or genomic imprinting. In cancer, tumor cells are characterized by global hypomethylation but locus-specific hypermethylation of promoter-associated CGI, resulting in gene silencing. MicroRNAs (miRNAs) are short, non-coding RNA sequences of 18–25 nucleotides, which can repress the translational of multiple protein-coding mRNAs by sequence-specific binding to the 3′untranslated region. Depending on the genes targeted, miRNA can be tumor suppressive if an oncogene is repressed, or it can be oncogenic when a tumor suppressive gene is repressed. Recently, aberrant methylation of tumor suppressive miRNAs has been reported in different types of cancers including lymphomas. Herein, we review the recent literature of methylation of tumor suppressive miRNAs in different histopathologic subtypes of lymphomas, and discuss its potential diagnostic, prognostic, and therapeutic significance.

## Lymphoma

Lymphoma results from neoplastic proliferation of lymphocytes. According to the World Health Organization classification, it can be broadly classified into Hodgkin and non-Hodgkin lymphomas (NHL; Swerdlow et al., 2008). Hodgkin lymphoma is characterized by CD30+ve Reed Sternberg cells with variable degree of reactive cellular infiltrate or sclerosis. NHLs are more heterogeneous, and are generally classified by the cell lineage, histological pattern, morphology, immunophenotype, or maturity of lymphoma cells. Based on the lineage of lymphoma cells, NHLs can be classified as B-, T-, or natural killer (NK)-cell lymphomas. For B- and T-cell lymphoma, they are further classified as precursor B- or T-cell lymphoid neoplasms, or mature B- or T-cell neoplasms (Chan, 2001; Au et al., 2005; Table 1). NHLs often present as nodal enlargements. However, extranodal sites such as the bone marrow, liver, spleen, mediastinum (Chim et al., 1996), heart (Chim et al., 1997), central nervous system, testis, breast, and the gastrointestinal tract can be involved. Moreover, some NHLs present primarily as extranodal disease. A prominent example is marginal zone B-cell lymphoma (MZBCL) of the stomach, which is an extranodal MZBCL of mucosa-associated lymphoid tissue (MALT) frequently associated with *Helicobacter pylori* infection of the gastric mucosa (Chan, 2001). Interestingly, in *H. pylori*+ve MALT lymphoma of the stomach, eradication of *H. pylori* infection alone may lead to resolution of gastric lymphoma.

**Table 1:** **Major types of mature B-, T-, and NK-cell lymphomas**.

**MATURE B-CELL NEOPLASMS**
Chronic lymphocytic leukemia/small lymphocytic lymphoma
Follicular lymphoma
Extranodal marginal zone lymphoma of mucosa-associated lymphoid tissue (MALT lymphoma)
Nodal marginal zone lymphoma
Splenic marginal zone lymphoma
Lymphoplasmacytic lymphoma
Mantle cell lymphoma
Plasma cell neoplasms
Burkitt lymphoma
Diffuse large B-cell lymphoma (DLBCL), NOS
Primary mediastinal (thymic) large B-cell lymphoma
DLBCL associated with chronic inflammation
B-cell lymphoma, unclassifiable, with features intermediate between DLBCL and Burkitt lymphoma
B-cell lymphoma, unclassifiable, with features intermediate between DLBCL and classical Hodgkin lymphoma
T-cell/histiocyte-rich large B-cell lymphoma
Intravascular large B-cell lymphoma
Plasmablastic lymphoma
Primary effusion lymphoma
**MATURE T- AND NK-CELL NEOPLASMS**
T-cell prolymphocytic leukemia
T-cell large granular lymphocytic leukemia
Peripheral T-cell lymphoma, NOS
Angioimmuoblastic T-cell lymphoma
Anaplastic large cell lymphoma (ALCL), ALK-positive
Anaplastic large cell lymphoma (ALCL), ALK-negative
Extranodal NK/T-cell lymphoma, nasal type
Aggressive NK-cell leukemia
Chronic lymphoproliferative disorders of NK-cells
EBV-positive T-cell lymphoproliferative disorders of childhood
Adult T-cell leukemia/lymphoma
Enteropathy-associated T-cell lymphoma
Hepatosplenic T-cell lymphoma
Subcutaneous panniculitis-like T-cell lymphoma
Mycosis fungoides
Sezary syndrome

Mature B-cell lymphomas can often be conceptually grouped by the putative maturation ontogeny of the neoplastic cells. Lymphomas arising from transformation of germinal center B-cells, which are CD10+ve, include follicular lymphoma (FL), Burkitt’s lymphoma (BL), and some diffuse large B-cell lymphoma (DLBCL). DLBCL is the most common form of mature B-cell lymphoma, comprising about 30% of all NHL. Despite its clinical aggressiveness, with combination chemotherapy, about half of the patients may be cured. On the other hand, small B-cell lymphomas comprise FL, small lymphocytic lymphoma (SLL), MZBCL, BL, lymphoplasmacytic lymphoma, and mantle cell lymphoma (MCL). FL is one form of small B-cell lymphoma prevalent in the Western population, accounting for about 20% of NHL. FL is characterized by the presence of *t*(14;18) with upregulation of BCL2, thereby conferring survival benefit to the lymphoma cells. It is an indolent disease of the elderly, and often presents with nodal and advanced stage disease. SLL is the nodal counterpart of chronic lymphocytic leukemia (CLL), and the tumor B-cells express dually CD5 and CD23 in addition to pan-B-cell antigens, CD19, and CD20. MZBCL, another form of indolent, small B-cell lymphoma, may occur as a nodal or splenic lymphoma in addition to the extranodal presentation described above.

On the other hand, despite being a form of small B-cell lymphoma, BL is an extremely aggressive lymphoma, characterized histologically by a starry sky appearance with literally all tumor cells simultaneously engaged in cell proliferation as evidenced by the almost 100% Ki67 immunoreactivity, and cytogenetically by *t*(8;14) in the majority of cases, which leads to upregulation of the *MYC* oncogene at 8q24. BL was first discovered in Africa as an extranodal lymphoma with a high proliferation rate associated with Epstein–Barr virus (EBV) infection. Subsequently, sporadic BL, often with extranodal presentation, had been diagnosed in other parts of the world.

Mantle cell lymphoma, once thought to be an indolent small B-cell lymphoma, is clinically moderately aggressive. It is characterized by *t*(11;14), leading to the uniform upregulation of cyclin D1 (Chim et al., 1998). MCL often presents with advanced disease with involvement of the lymph nodes and frequent extranodal involvement including spleen, blood, and bone marrow. (Chim et al., 2003a) With standard therapy, MCL is associated with a short remission duration with a median overall survival of 4–5 years (Vose, 2012).

It is notable that T-cell lymphoma is much less frequent than B-cell lymphoma, and depending on the geographic location, it comprises only 8–15% of all NHLs (Au et al., 2005). Anaplastic large cell lymphoma (ALCL), peripheral T-cell lymphoma, unspecified, and angioimmunoblastic T-cell lymphoma (AITL) are the most common forms of mature PTCL (Au et al., 2005). Most forms of mature T-cell lymphomas are nodal lymphomas. Of these, the pathology of PTCL, unspecified, were variably described as T-zone lymphoma, lymphoepithelioid cell lymphoma, pleomorphic T-cell lymphoma, small, medium, or large sized types, and T-immunoblastic lymphoma; so that there was a broad morphologic spectrum and immunophenotypic profiles. On the other hand, AITL is a disease of the elderly, with the majority being nodal and advanced stage at presentation. Pathologically, there was a polymorphic lymphomatous infiltrate admixed with reactive lymphocytes, eosinophils, plasma cells, and histiocytes, with arborizing high endothelial venules. Systemic manifestations includes hepatosplenomegaly, ascites, proteinuria, and high fever (Chan, 2001; Au et al., 2005). Finally, in ALCL, lymphoma cells are large and pleomorphic, expressing CD30 in addition to T-cell markers. ALCL can further be classified by the expression of the anaplastic lymphoma kinase (ALK) into ALK-positive and ALK-negative subtupes. In ALK-positive ALCL, ALK is often activated as a result of reciprocal translocation of the *ALK* gene with another partner gene, with *t*(2;5) being the prototype that results in fusion of the nucleophosmin gene with ALK. ALK-positive ALCLs carry a favorable prognosis compared with ALK-negative ALCLs.

Extranodal NK/T-cell lymphoma, nasal type, is derived putatively from NK-cells which are often CD56-positive. The majority of NK-cell lymphomas present primarily in the nasal area and upper aerodigestive tract, and is referred to clinically as nasal NK-cell lymphomas (Chim et al., 1999, 2004c). Occasionally, NK-cell lymphomas can arise in extranasal sites, involving the skin, gastrointestinal tract, salivary glands, and testis, and are referred to as non-nasal NK-cell lymphomas. Rarely, NK-cell lymphomas can present in a leukemic phase, and are referred to as NK-cell leukemia (Kwong, 2005). NK-cell neoplasms are rare but aggressive lymphomas uniformly associated with EBV infection. Patients with nasal NK-cell lymphoma often present with fever and nasal symptoms, and locally destructive lesions. Despite the initial nasal presentation, lymphoma cells may eventually disseminate to other extranodal sites, which often are the presentation sites of non-nasal NK-cell lymphomas. Finally, NK-cell leukemia presents with pancytopenia, bone marrow failure, and is almost always lethal.

## miRNA

With complete sequencing of the human genome, an increasing amount of non-coding RNAs (ncRNAs) are unveiled and shown to work in concert with the protein-coding gene network (Human Genome Sequencing, 2004; Lander, 2011). miRNA, which is a widely studied subclass of short ncRNAs with a length of 18–25 nucleotides, leads to translational repression of protein-coding genes via sequence-specific binding of its seed region to the 3′untranslated region (UTR) of its target protein-coding genes. Currently more than 1500 miRNAs have been discovered. Interestingly, many miRNAs have been implicated in carcinogenesis (Kozomara and Griffiths-Jones, 2011). Based on the genomic location, miRNA can be classified as intergenic and intragenic. Intergenic miRNA is transcribed from non-coding region in between protein-coding genes, whereas intronic miRNA is encoded in the intron of, and mostly transcribed in parallel with, its host protein-coding gene (Lopez-Serra and Esteller, 2012).

**Figure 1:**
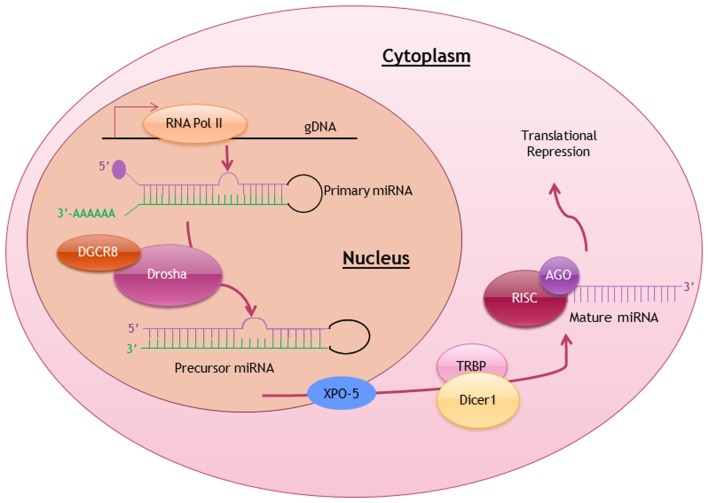
**Biosynthesis and posttranscriptional processing of miRNA**. Schematic diagram summarizing the biosynthetic pathway and processing of miRNA to produce a mature miRNA ready to exert it function. RNA Pol II, RNA polymerase II; DGCR8, DiGeorge syndrome critical region gene-8; XPO-5, exportin-5; TRBP, TAR-binding protein; RISC, RNA-induced silencing complex; AGO, Argonaute.

Similar to the transcription of protein-coding genes, most miRNA genes are also transcribed by RNA polymerase II with the inclusion of 5′ cap structure and 3′ polyadenylated tail in each of the primary miRNA (pri-miRNA) transcript (Lee et al., 2004). Pri-miRNAs, ranging from 100 to 1000 nucleotides in length, are then processed by a ribonuclease-III DROSHA complex with DGCR8 (DiGeorge Syndrome Critical Region Gene-8) into a stem-looped precursor miRNA (pre-miRNA; Gregory et al., 2004). These intermediates pre-miRNAs are exported via Ran-GTP-dependent exportin-5 (XPO-5) into the cytoplasm, where these pre-miRNA stem-loops are further processed into mature miRNA duplex (Yi et al., 2003). Eventually a single-stranded mature miRNA is produced, ready to function when it is loaded onto the DICER1-TAR RNA-binding protein-containing RNA-induced silencing complex (RISC; Liu et al., 2004). The biosynthesis and processing of miRNA is summarized in Figure 1.

Several functional mechanisms of miRNA have been described. However, the specific binding of miRNA seed region to the 3′UTR of mRNA via imperfect complementarity (Lopez-Serra and Esteller, 2012) has attracted much attention. Such binding leads to blockage of protein translation and hence translational silencing of the target protein-coding gene. Depending on the silenced target gene, miRNA can also be divided into tumor suppressive and oncogenic miRNA. A tumor suppressive miRNA targets an oncogene while an oncogenic miRNA targets a tumor suppressive gene. In lymphoma or solid cancers, in which viral infections is involved in carcinogenesis, potentially oncogenic miRNA may be derived from the integrated viral genome (Lin et al., 2010; Brown et al., 2012). Based on miRNA array studies, a multitude of tumor suppressive miRNAs has been shown to be downregulated in different types of lymphomas (Craig et al., 2011; Dejean et al., 2011; Iqbal et al., 2012). The primary objective of this article is to review the intricate role of tumor suppressive miRNAs that are silenced in lymphoma, and to discuss the emerging role of DNA methylation in mediating the expression of these miRNAs.

## DNA Methylation-Mediated Gene Silencing

DNA methylation is an important epigenetic mechanism for cells to maintain their normal gene expression patterns. Gene methylation is crucial for genomic imprinting and X-chromosome inactivation for correct tissue and organ development (Reik and Walter, 2001; Kaneda et al., 2004). DNA methylation occurring on CpG dinucleotides is mediated by DNA methyltransferases (DNMT)1, 3A, and 3B, which add a methyl group to the C5 carbon of the cytosine residue in the CpG dinucleotides (Okano et al., 1998). CpG rich regions, known as CpG islands (CGI), can be localized at the promoter region of a protein-coding or miRNA gene. CGI methylation results in a silencing effect on the associated protein-coding and miRNA genes. To determine the roles of CGIs in regulating gene expressions, genome-wide studies have been conducted to examine the methylation status of CpGs across the human genome in both normal and cancer cells. An account of methylation-mediated genomic imprinting and cancer-related transcriptional silencing is given below (Shen et al., 2007; Choi et al., 2010; Sandoval et al., 2011).

## Methylation of Tumor Suppressive miRNA in NHL

**Figure 2:**
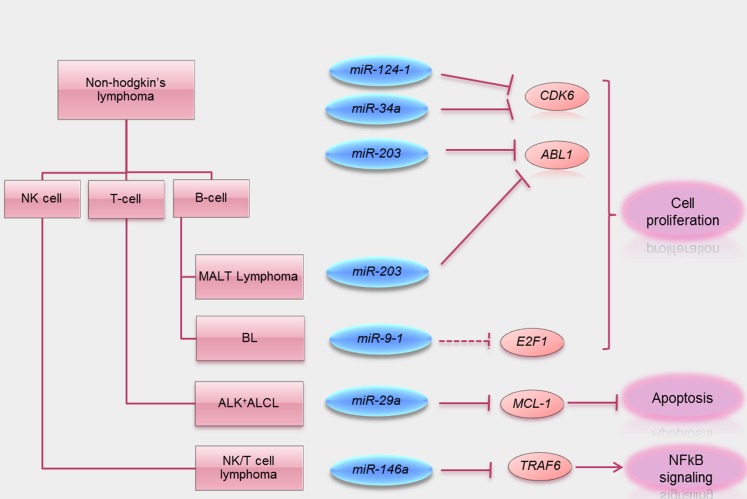
**Summary of tumor suppressive functions of miRNA methylated in lymphoma**. Schematic visualization summarizing miRNA methylated in different subtypes of lymphomas known direct target oncogenes (solid line) and putative target oncogenes (dotted lines) silenced by these tumor suppressive miRNA. A key tumor suppressive function of these miRNA is also summarized.

All articles with the keywords “lymphoma, DNA methylation and miRNA” obtained from a search of the PubMed were reviewed. They can be classified into two groups. The first group comprised mechanistic studies of miRNA methylation in special subtypes of NHL including NK-cell lymphoma (pertaining to *miR-146a*), gastric MALT lymphoma (*miR-203*), *t*(8;14)-negative BL (*miR-9*), and ALK-positive ALCL (*miR-29a*). The second group comprised studies of the impact of miRNA methylation in a panel of B-, T-, or NK-cell lymphomas (Table 2 and Figure 2).

**Table 2:** **Methylated tumor suppressive miRNA common in both lymphoma and other hemic cancers/solid tumors**.

miRNA	Hematological cancer	Solid tumor type	Chromosome location
*miR-124-1*	ALL (Agirre et al., 2012), AML, CLL, myeloma (Wong et al., 2011)	Cervical (Wilting et al., 2010), colorectal (Lujambio et al., 2007), liver (Furuta et al., 2010)	8p23.1
*miR-203*	NHL, MALT lymphoma, DLBCL (Chim et al., 2011b)	Liver (Furuta et al., 2010), cervical (Botezatu et al., 2011)	14q32.32
*miR-34a*	NHL, CLL, myeloma (Chim et al., 2010)	Melanoma, prostate (Lodygin et al., 2008)	1p36.22
*miR-9-1*	BL (Onnis et al., 2010)	Colorectal (Bandres et al., 2009), breast (Lehmann et al., 2008), pancreatic (Omura et al., 2008)	1q22

### miRNA methylation in special NHL subtypes

#### *miR-146a* methylation in NK/T-cell lymphoma

Promoter methylation of *miR-146a* was described in a study of primary NK/T-cell lymphoma, which was associated with downregulation of *miR-146a* (Paik et al., 2011). Over-expression of *miR-146a* was then shown to inhibit lymphoma cell proliferation and induce apoptosis, thereby demonstrating its tumor suppressor properties. In addition, based on luciferase assay, over-expression of *miR-146a* was shown to lead to inhibition of the NFκB pathway, due to binding to and hence blockage of NFκB responsive elements, thereby illustrating a role of *miR-146* on NFκB signaling. On the other hand, TNF receptor-associated factor 6 (TRAF6) has been shown to transactivate the NFκB pathway by promoting proteasomal degradation of IκB, the inhibitor of NFκB. By bioinformatic search, TRAF6 was found to possess *miR-146a* binding sites at the 3′UTR, to which *miR-146a* binding has been demonstrated (Starczynowski et al., 2010). Besides, the authors showed that downregulation of TRAF6 was achieved by over-expression of *miR-146a*, consistent with the notion that TRAF6 is also a target of *miR-146a* (Paik et al., 2011). Moreover, downregulation of TRAF6 by siRNA led to inhibition of the NFκB pathway and consequent downregulation of anti-apoptotic BCL2, consistent with the notion that *miR-146a* regulated NFκB pathway via regulating TRAF6 expression. Furthermore, *miR-146a* was shown to confer chemosensitivity of NK lymphoma cells to etoposide, a chemotherapy active in NK/T-cell lymphoma. Finally, patients with higher *miR-146a* expression had better survival than those with low expression. Therefore, *miR-146a* is a putative tumor suppressive miRNA, acting via repression of TRAF6, and hence downregulation of NFκB signaling. Its frequent hypermethylation in NK/T-cell lymphoma might be of prognostic significance.

#### *miR-203* methylation gastric MALT lymphoma

A recent study of miRNA expression in *H. pylori*-positive gastric MALT lymphoma showed differential expression of miRNA as compared with normal tonsils. In particular, *miR-203* was shown to be downregulated in lymphoma (Craig et al., 2011), whereas the adjacent inflammatory but non-lymphoma tissues with chronic gastritis had higher expression levels. This was biologically relevant, as *miR-203* expression was inversely related to the expression of the *miR-203* target gene *ABL1*. Furthermore, *miR-203* was unmethylated in normal tonsil, minimally methylated in gastritis tissue, but completely methylated in gastric MALT or DLBCL of stomach, which was derived from transformation of MALT lymphoma. Therefore, *miR-203* methylation appeared specific to gastric lymphoma, which might have started with chronic gastritis. The tumor suppressive function of *miR-203* was demonstrated in a mouse model, in which gastric MALT lymphoma developed after prolonged *H. felis* infection (Craig et al., 2011). In this model, *miR-203* was highly expressed in normal marginal zone B-cells, modestly expressed in gastritis tissue and under-expressed in gastric MALT lymphoma tissue with corresponding upregulation of its target *ABL1* proto-oncogene. Over-expression of *miR-203* led to decreased *ABL1* mRNA expression and a blockage of *Helicobacter* antigen-dependent cell proliferation. Finally, treatment with a tyrosine kinase inhibitor of imatinib that targeted *ABL1* led to regression of gastric tumors (Craig et al., 2011). Therefore, *miR-203* was shown to be a tumor suppressive miRNA targeting *ABL1*, and *miR-203* methylation might be important in pathogenesis of MALT lymphoma.

#### *miR-29a* methylation in ALK+ve ALCL

A recent study showed that the expression of *miR-29a* was suppressed by promoter methylation in ALK-positive ALCL (Desjobert et al., 2011). Consistent with this observation, the myeloid cell leukemia 1 (MCL1) gene, a known target of *miR-29a*, was highly expressed in ALK-positive ALCL, whereas over-expression of *miR-29a* resulted in downregulation of MCL1 (Xiong et al., 2010; Desjobert et al., 2011). Interestingly, *miR-29a* expression could be restored either with knockdown of ALK or its inhibition, suggesting that ALK is involved in *miR-29a* methylation. Furthermore, knockdown of ALK or STAT3, known targets of *miR-29a*, led to downregulation of DNMT1 and DNMT3 (Desjobert et al., 2011). Therefore, ALK might repress *miR-29a* via upregulation of DNMT1 and hence hypermethylation of the *miR-29a* promoter. Finally, in a xenograft tumor model of ALK-positive ALCL, tumors with over-expression of *miR-29a* were much smaller that those without231. These observations strongly suggested that *miR-29a* was a tumor suppressive miRNA hypermethylated in ALK-positive ALCL.

#### *miR-9-1* methylation in *t*(8;14)-ve BL

The expression of mature *miR-9* from the 3′ arm of the three *miR-9* precursors *miR-9-1*, *miR-9-2*, *miR-9-3* (derived from different genomic loci) was low in *t*(8;14)-negative BL, as compared with DLBCL or *t*(8;14)-positive BL, thereby suggesting that *miR-9-3p* might be of pathogenetic significance in BL without *t*(8;14; Onnis et al., 2010). The low expression of *miR-9-3p* was found to be due to DNA hypermethylation of one of the three genomic loci, *miR-9-1*. The result was an upregulation of E2F1, a putative target of *miR-9-3p*. Since E2F1 is also a transcriptional target of *c-MYC*, *miR-9-1* methylation with upregulation of E2F1 might be similar to the biological consequence of *t*(8;14), where *c-MYC* dysregulation might also result in excessive transcriptional activation of E2F1, leading in both instances to unchecked cellular proliferation (O’Donnell et al., 2005).

### miRNA hypermethylated in B-, T-, or NK-cell lymphomas

Based on a candidate miRNA approach, *miR-124-1*, *miR-203*, and *miR-34a* methylation in B-, T-, and NK-cell lymphomas have been studied.

#### *miR-124-1* methylation in NHL

*miR-124-1* methylation has been studied in a panel of primary samples of hematological cancers including acute myeloid leukemia (AML), acute lymphoblastic leukemia (ALL), chronic myeloid leukemia (CML), CLL, multiple myeloma (MM), and B-cell, T-cell, and NK-cell NHLs (Wong et al., 2011). In primary samples at diagnosis, *miR-124-1* methylation was absent in CML but detected in 2% each of MM at diagnosis and relapse/progression; 5% of ALL; 15% of AML; 14% of CLL, and 58.1% of NHL. Amongst lymphoid malignancies, *miR-124-1* was preferentially methylated in NHL than MM, CLL, or ALL. In primary lymphoma samples, *miR-124-1* was preferentially hypermethylated in B- or NK/T-cell lymphomas, in which *miR-124-1* expression correlated inversely with *miR-124* expression, thereby confirming miRNA silencing in association with miRNA methylation (Wong et al., 2011). As chromosome 8p23 is found commonly lost in certain subtypes of B-cell NHL; frequent *miR-124-1* methylation in NHLs might pose an alternative mechanism of *miR-124-1* inactivation, which may collaborate with *miR-124-1* deletion to fulfill the two-hit Knudson’s hypothesis (Knudson, 2001; Martinez-Climent et al., 2001; Callet-Bauchu et al., 2005).

#### *miR-203* methylation in NHL

Another study reported the status of *miR-203* methylation in 150 patients with various hematological cancers (Chim et al., 2011b). In primary samples, *miR-203* methylation was detected in 5.0% of ALL, 10.0% of AML, 42.0% of CLL, and 38.8% of NHL (including 60.0% of NK, 40.9% of B-cell, and 23.5% of T-cell NHL, and hence *miR-203* was more frequently hypermethylated in lymphoid than myeloid malignancies. Amongst the NHL samples studied, *miR-203* was methylated in 40.9% of B-cell NHL, 23.5% of T-cell NHL, and 60% of NK/T-cell lymphoma. However, there was no correlation between *miR-203* methylation with clinical parameters including age, gender, nodal/extranodal presentation, or Ann Arbor stage. Interestingly, in the patients in which other miRNAs were concomitantly studied, *miR-203* methylation was found to be associated with methylation of *miR-124-1*, *miR-34a*, and *miR-196b*. As both *miR-124* and *miR-34a* target CDK6, the simultaneous methylation of these miRNAs might contribute to constitutive activation of certain oncogenic pathways. Alternatively, inactivation of multiple tumor suppressive miRs targeting multiple pathways might be involved in lymphomagenesis. For instance, *miR-203* methylation might contribute to tumor cell survival through upregulation of *ABL1*, and *miR-124-1* methylation to proliferative advantage by upregulation of CDK6 (Martinez-Climent et al., 2001; Craig et al., 2011; Wong et al., 2011).

#### *miR-34a* methylation in NHL

Similarly, *miR-34a* methylation has been studied in a panel of hematological cancers including 20 ALL, 20 AML, 11 CML, 50 CLL, 55 MM, and 32 NHL patients. In primary samples at diagnosis, *miR-34a* methylation was detected in 4% of CLL, 5.5% of MM, and 18.8% of NHL at diagnosis, but not in ALL, AML, and CML (Chim et al., 2010). Amongst lymphoid malignancies, *miR-34a* was preferentially methylated in NHL, in particular NK/T-cell lymphoma. The findings suggested that mechanisms controlling the expression of a miRNA are cancer dependent, although miRNA might exert its tumor suppressive function in a similar mechanistic action in lymphoma of different subtypes.

## Discussion

Based on the above summary, a few points are worth further discussion.

Firstly, while mechanistic studies have been performed to illustrate the role of miR methylation in the pathogenesis of certain lymphoma subtypes (such as methylation of miR-146a in NK-cell lymphoma, miR-230 in gastric MALT lymphoma, and miR-29a in ALK+ve ALCL), it is likely that multiple miRs may be hypermethylated in any single lymphoma subtype. For instance, miR-203 have been shown to be hypermethylated in a multitude of hematological cancers especially CML and Ph+ve ALL (Bueno et al., 2008), and hence not restricted to gastric MALT lymphoma. This was further illustrated by our work that showed miR-203 methylation in multiple lymphoid malignancies including B-CLL, and B-cell, T-cell and even NK-cell lymphoma (Chim et al., 2011b). Therefore, multiple miRs are likely hypermethylated in a lymphoma subtype, and hence a lymphoma type-specific miR methylation profiles.

Secondly, miR-146a was shown hypermethylated in NK-cell lymphoma associated with upregulation of NFkB pathway (Paik et al., 2011). However, based on a candidate gene approach, multiple miRs have been shown to be hypermethylated in NK-cell lymphoma too. For instance, miR-34a, -203, and -124-1 were frequently methylated in NK-cell lymphomas (Chim et al., 2010, 2011a; Wong et al., 2011). Moreover, a recent miR array study of nasal NK-cell lymphoma showed that multiple miRs were downregulated in NK-cell lymphoma including miR-101, miR-26a, miR26b, miR-28-5, and miR-363 (Ng et al., 2011). Therefore, methylation may occur in multiple miR promoter-associated CGI, regulating multiple signaling pathways including NFkB.

Thirdly, while some of these reported miRNAs, such as miR-9-1, 124-1, and -203, are embedded in classical CGI with proven promoter activities, some miRNAs were reported to be hypermethylated at scanty CpG sites upstream of the respective miRNA. For instance, miR-29a was postulated to be regulated by a stretch of 5 CpG sites at 1.1 kb upstream of its precursor sequence (Desjobert et al., 2011). Similarly, miR-146a was suggested to be regulated by two sparsely separated CpG dinucleotides immediately upstream of its precursor sequence, instead of the defined primary sequence (Paik et al., 2011). Last but not least, as DNMT has been shown to be the translational target of miR-29 family miRs, it would be interesting to see if restoration of miR-29a in hypermethylated cell lines may lead to re-expression of methylation-silenced tumor suppressor genes in addition to downregulation of DNMT family genes. For instance, miR-29b restoration in AML cells led to global DNA hypomethylation, resulting in re-expression of CDKN2B and estrogen receptor 1 (ESR1) through promoter DNA hypomethylation (Garzon et al., 2009). Moreover, in lung cancer, miR-29 family miRs (29a, 29b, and 29c) directly target both DNMT3A and -3B, and over-expression of miR-29s in lung cancer cell lines restores normal patterns of DNA methylation, induces re-expression of methylation-silenced tumor suppressor genes and inhibits tumorigenicity *in vitro* and *in vivo* (Fabbri et al., 2007).

In conclusion, there is much to be learned of miR methylation in NHL regarding the biology, pattern and its role in lymphomagenesis.

## Conclusion and Future Perspective

In conclusion, the pathogenetic role of miRNAs methylation in lymphomagenesis is just beginning to be unraveled. In contrast, there is considerable data to show that inactivation of tumor suppressor genes by DNA methylation plays important roles in the pathogenesis and prognostification of hematological malignancies (Chim et al., 2001a,b, 2003b, 2004a,b, 2005a,b). For instance, methylation of retinoic acid receptor alpha (RARA), PTPN6, and DAPK genes has been implicated in pathogenesis of AML, myeloma, and CLL respectively (Reik and Walter, 2001; Chim et al., 2002, 2006; Kaneda et al., 2004). Moreover, methylation of CDKN2B and WNT inhibitory factor 1 (WIF1) has been shown to be an independent prognostic factor predicting inferior disease-free survival in acute promyelocytic leukemia (Chim et al., 2001b, 2003b, 2005a). Therefore, future studies will help to confirm the pathogenetic and prognostic impact of miRNA methylation in NHLs.

DNA methylation can be reversed by demethylating agents 5-azacytidine or decitabine, and therapeutic benefits have been demonstrated in patients with myelodysplastic syndrome (Garcia-Manero and Fenaux, 2011). Besides, histone deacetylase inhibitors including vorinostat and panobinostat (Duvic and Vu, 2007), have been shown to be effective for some forms of lymphoma such as mycosis fungoides. As 5-azacytidine or decitabine may act synergistically with histone deacetylase inhibitors to enhance re-expression of genes silenced by hypermethylation, this strategy might also be applied to miRNA hypermethylation. Finally, there is hope that downregulated tumor suppressor miRNA in cancer may be restored by the use of miRNA mimics (Henry et al., 2011), which can form a partially double-stranded RNA mimicking endogenous pre-miRNA to be processed into an active miRNA molecule (Calin and Croce, 2009). miRNA can be delivered to the tumor in the form of an oligonucleotide mimic or by expressing the miRNA in the cancer using a gene vector (Henry et al., 2011).

## Conflict of Interest Statement

The authors declare that the research was conducted in the absence of any commercial or financial relationships that could be construed as a potential conflict of interest.
